# Efficacy and Safety of Kahook Dual Blade Goniotomy and Trabecular Micro-Bypass Stent in Combination with Cataract Extraction

**DOI:** 10.3390/biomimetics10100691

**Published:** 2025-10-14

**Authors:** Kevin Y. Wu, Shu Yu Qian, Lysa Houadj, Michael Marchand

**Affiliations:** 1 Department of Surgery, Division of Ophthalmology, University of Sherbrooke, Sherbrooke, QC J1G 2E8, Canada; 2 Faculty of Medicine, University of Sherbrooke, Sherbrooke, QC J1H 5H3, Canada

**Keywords:** glaucoma, intraocular pressure, microinvasive surgery, Kahook Dual Blade, iStent

## Abstract

In recent years, rapid advancements in glaucoma research have led to the development of more effective treatments of this chronic and irreversible condition. Of these, Kahook Blade Dual (KDB) goniotomy and second-generation trabecular micro-bypass stent (iStent) are two novel biomimetic procedures which have designs inspired by the eye’s natural drainage mechanisms. In this retrospective study, we evaluated the safety and effectiveness of both surgeries by including 176 eyes from 110 patients: 142 eyes in the iStent group and 34 in the KDB group. The primary outcomes of this study were the proportions of patients in each group attaining a 20% reduction in IOP and a post-operative IOP < 19 mmHg. At the last follow-up, a 20% reduction in IOP was achieved by 67% of iStent inject patients and 50% of KDB patients (*p* = 0.07). The iStent group also showed a higher proportion of patients reaching an IOP of less than 19 mmHg (81% vs. 71% in the KDB group, *p* = 0.13). The number of medications did not decrease in either group from pre-op to the last follow-up. The KDB group had more failures (29.4% vs. 4.2%) and a significantly higher adverse event rate than the iStent inject group (47.1% vs 12.0%).

## 1. Introduction

Glaucoma is one of the leading causes of irreversible blindness worldwide [1]. Currently, the global prevalence is estimated at 3.5% with a projected increase of 50% over the next twenty years [2]. This chronic disease requires persistent lifelong treatment with a spectrum of therapeutic options like medications, laser procedures, and surgeries. The common goal of these intraocular pressure (IOP)-lowering therapies is to prevent the retinal ganglion cell loss that results in visual field defects [3]. While the concept of IOP as the only modifiable risk factor has recently been challenged, the reduction in this parameter is nonetheless the cornerstone to slowing disease progression [4,5]. Microinvasive glaucoma surgery (MIGS) is a growing class of procedures used to lower IOP that often exploit biomimetic principles to enhance the eye’s natural outflow pathways while minimizing damage to surrounding tissue [6]. Since its emergence over the last two decades, MIGS has shifted the treatment paradigm by offering the possibility of earlier surgical interventions in the course of the disease [7]. Typically, initial glaucoma therapy consists of eye drops, laser trabeculoplasty, or both. When these treatments are insufficient, incisional surgeries are required. However, these surgeries result in labile post-operative IOPs, need prolonged recovery times, and risk serious short- and long-term complications. These interventions are thus generally reserved for severe cases and are delayed for those with mild to moderate glaucoma. As a result, a gap in treatment was present for mild to moderate glaucoma patients with uncontrolled IOP despite medical and laser therapy. MIGS strives to fulfill this need. All MIGS interventions work by increasing aqueous humor outflow from the anterior chamber, either by diverting the aqueous humor to the suprachoroidal or subconjunctival space or by directly accessing Schlemm’s canal. These procedures, defined by an ab interno conjunctival-sparing microincision, provide an improved safety profile compared to traditional glaucoma surgery and offer a rapid recovery [8]. These features favor pairing MIGS with phacoemulsification (phaco) for patients with co-existing glaucoma and cataract. Furthermore, a surgical intervention lowers the medication burden and its associated drawbacks such as side effects and costs [9]. Surgical treatment has also shown smaller IOP fluctuations than drug treatment [10]. Due to the infancy of many MIGS interventions, there are currently limited robust comparative studies evaluating the efficacy and safety of these approaches.

Two MIGS procedures, goniotomy using the Kahook Dual Blade (KDB, New World Medical Inc., Rancho Cucamonga, CA, USA) and the iStent inject (Glaukos Corp., San Clemente, CA, USA), both bypass the trabecular meshwork (TM) by different biomimetic mechanisms. KDB is a Food and Drug Administration (FDA) class I device, the usage of which is not limited by type or severity of glaucoma. The device enters the anterior chamber, pierces the TM, and advances along its length to excise a TM segment of approximately 90–120°. The inbuilt ramp at the distal end of the device lifts the TM tissue, and two parallel blades excise the tissue and the inner wall of Schlemm’s canal, thereby preserving the integrity of the canal and simply enhancing the eye’s aqueous outflow system [11]. Additionally, this procedure improves fluid dynamics via an anatomically precise approach that maintains the eye’s natural architecture. The excised tissue is removed, thus reducing the risk of scarring and potential failure of the surgery [12]. Since the introduction of KDB in 2015, a rising number of studies have demonstrated its efficacy and safety as a standalone procedure and with phacoemulsification in reducing IOP and the medication burden [13,14,15,16]. In clinical studies, KDB alone lowered IOP by 24–36% and reduced medication usage by 32–40% at 6 months, and when combined with phacoemulsification, it reduced IOP by 14–33% and medications by 40–63% at 6 months [14,15,16,17]. Goniotomy using KDB is effective in mild to advanced primary open-angle glaucoma (POAG) and has also been performed successfully in congenital glaucoma, secondary glaucoma, and primary angle-closure glaucoma (PACG) [16,18,19].

The iStent inject Trabecular Micro-Bypass System Model G2-M-IS contains two preloaded heparin-coated titanium stents delivered by a single injector. This device is designed to re-establish flow of the aqueous humor into Schlemm’s canal by targeting the juxtacanalicular part of the TM, which represents the maximal resistance to aqueous humor outflow in patients with POAG [20]. The stent’s head is inserted into Schlemm’s canal, and the rear flange resides in the anterior chamber. The thorax of the stent connects these two portions and is retained by the TM, thus creating a bypass through the TM and improving aqueous outflow through the natural physiologic pathway by mimicking the function of the canal’s endothelial lining [21]. These naturalistic features allow the optimization of fluid dynamics by imitating the eye’s physiological aqueous humor drainage. The FDA pivotal trial randomized 505 eyes in a 3:1 ratio to combined cataract surgery with iStent inject versus cataract surgery alone. The primary outcome was the proportion of eyes achieving a 20% reduction in washed-out IOP between baseline and 24 months. In that study, 76% of iStent inject eyes achieved a 20% reduction in IOP compared to only 62% of those receiving only cataract surgery. The reduction in unmedicated diurnal IOP was also significantly greater in the iStent inject arm at 7.0 mmHg than in the control group at 5.4 mmHg [22]. Subsequently, numerous studies have also demonstrated the efficacy and safety of the iStent inject as a standalone procedure and combined with cataract surgery for the treatment of POAG [22,23,24,25]. Such examples of recent breakthroughs in glaucoma research represent a shift away from traditional invasive surgeries and towards biomimetic tissue-sparing approaches [26].

Few studies have compared the surgical outcomes of the iStent inject with other MIGS interventions, and they mostly investigated the older first-generation iStent [27,28]. The literature comparing the first-generation iStent with KDB for POAG finds either comparable IOP decrease or superior results with KDB [29,30,31]. Reduction in the number of medications varies in these studies: some reporting no significant difference between the two procedures while others reporting a greater reduction with one or the other [29,31,32]. Although there is considerable literature on the surgical outcomes of KDB and iStent inject, there are few comparison studies for either of these interventions. A single study comparing KDB with iStent inject in POAG indicates KDB offering a more favorable IOP reduction than the iStent inject, but their results were not statistically significant [33]. The purpose of our study is to compare the efficacy and safety of excisional goniotomy performed with KDB versus the implantation of the second-generation iStent inject trabecular bypass device combined with phacoemulsification across the entire spectrum of glaucoma severity.

## 2. Materials and Methods

### 2.1. Participants

This study was a retrospective, single-center, observational, longitudinal case series that compared the efficacy and safety of two different types of microinvasive glaucoma surgery procedures (KDB goniotomy and a second-generation trabecular micro-bypass stent) in combination with cataract extraction. All patients that underwent these procedures between July 2021 and June 2023 inclusively at Hôtel-Dieu de Sherbrooke, QC, Canada, were included in the analysis. They were operated on by a single experienced surgeon (M.M.) who had performed at least 80 prior cases of iStent inject and KDB during his training period, which were not included in this current study.

Our inclusion criteria are as follows: patient having undergone either an iStent inject implantation or a goniotomy using KDB, having a clinical diagnosis of glaucoma defined as characteristic glaucomatous changes to the optic nerve head with or without visual field defects (ocular hypertension), 18 years of age or older, and having a minimum of 12 months of follow-ups. Those meeting the following criteria were excluded: complicated phacoemulsification surgery (vitreous loss, vitrectomy, intraocular lens implantation outside the lens capsule), presence of ocular comorbidities limiting potential post-operative visual gains (optic atrophy, neovascular age-related macular degeneration), inability to visualize nasal trabecular meshwork, elevated episcleral venous pressure, acute or chronic ocular inflammation, and previous history of failed MIGS on the same eye.

### 2.2. Study Design

Before surgery, the patient underwent a comprehensive evaluation, which involved reviewing their ophthalmic history, measuring their IOP with the Goldmann applanation technique (without stopping medication), assessing their visual acuity (VA) at 20 feet using the Snellen chart under standardized lighting conditions, conducting indirect gonioscopy, examining their anterior and posterior segments using non-dilated and dilated slit lamp bio-microscopy (with a 90D lens), and performing standard automatic perimetry using the Humphrey^®^ Field Analyzer 3 (Carl Zeiss Meditec Inc., Dublin, CA, USA). Snellen values were converted to a logarithm of the minimal angle of resolution (logMAR) for ease of interpretation during statistical analysis.

The indications for both surgeries were similar, and the most common reasons for surgery were suboptimal intraocular IOP or slow visual field progression, despite maximal tolerable glaucoma therapy, with or without previous selective laser trabeculoplasty. Another group of patients included those using more than two types of topical glaucoma drops without known intolerance who required additional IOP reduction and wished to reduce their medication load. The choice between iStent inject or KDB goniotomy was based on the surgeon’s preferences and a detailed discussion with the patient, during which informed consent was obtained in all cases.

The protocol of this study was approved by the Institutional Review Board of the Centre de recherche du CHUS (2023-4911) on 12 December 2022 and adhered to the tenets of the Declaration of Helsinki.

The surgical technique used for KDB goniotomy and iStent inject implantation followed the previously described procedure. For all phakic patients, a standardized routine of phacoemulsification and intraocular lens implantation was performed first. Following cataract surgery, 1% acetylcholine was administered into the anterior chamber (AC) to constrict the pupil, after which iStent implantation or KDB goniotomy was performed.

Patients were scheduled for routine follow-up visits, typically starting on day 1 after surgery and continuing at 2 weeks, 1 month, 3 months, 6 months, 9 months, 12 months, 15 months, 18 months, and 24 months. During these visits, the number and type of glaucoma medications were assessed, along with standard best-corrected visual acuity (BCVA), Goldmann IOP (without washout), indirect gonioscopy, and anterior and posterior segment (90D) slit lamp examination. Visual field examinations were conducted based on clinical needs.

Patients were typically given a post-operative topical therapy of 0.3% nepafenac once daily for 3 to 4 weeks, as well as topical preservative-free 0.1% dexamethasone 3 times daily and tapered over a standard 3-week course. After the surgery, the ophthalmologist continued to administer IOP-lowering medications, and the first decision to discontinue some of them was made 6 to 8 weeks after surgery.

### 2.3. Outcome Measures

The primary outcome measures for this study were pre- and post-operative IOPs, the number of patients with a >20% post-operative IOP reduction, and the number of patients with a post-operative IOP < 19 mmHg at the last follow-up. These chosen cut-offs align with previous studies that focused on the same surgical techniques. In particular, the value of 20% originates from the Ocular Hypertension Treatment Study and is the reference value used in glaucoma treatment studies [34].

In addition to the primary outcome measures, this study also evaluated several secondary outcomes. These included the average IOP at different follow-up times, the average IOP reduction achieved by the surgical procedures, and the number of IOP-lowering medications needed after surgery. Adverse events related to the procedures were monitored and recorded. Failure criteria, which included the need for IOP-lowering surgery during the follow-up period, were evaluated. Finally, the study also assessed BCVA at the last follow-up.

### 2.4. Statistical Analysis

Our analyses utilized descriptive statistics for the complete sample as well as separately for each arm. Categorical data were presented with frequencies and percentages. Socio-demographic comparisons between arms were evaluated using the Student’s *t*-test for continuous data (Mann–Whitney U test) and chi-square tests (Fisher’s exact test) for categorical variables. Normality was assessed visually with histograms. Given the longitudinal design, missing values at different time points were handled using maximum likelihood estimation of mixed models. All tests were two-tailed, with a significance level of 5%. Statistical analyses were performed using R v.4.3.0 (R Core Team, Vienna, Austria).

Success rates at the last follow-up were compared between groups using the chi-square test. For trajectories, linear mixed models were used to assess the evolution of arms over time, as the dependent variables consisted of a continuous variable collected at various time points. A random intercept was added at the patient level to account for the correlation of repeated measures, and fixed effects were considered for arm, time, and their interaction. A multivariable model was also presented when adjusting for potential confounding factors (age, gender, lens status, type of glaucoma, glaucoma medication pre-op, and severity). The results were reported as model coefficients (mean differences) along with 95% confidence intervals. Assumptions of residual normality and homoscedasticity were assessed visually using appropriate diagnostic plots.

## 3. Results

### 3.1. Patient Characteristics

A total of 88 patients (142 eyes) were included in the iStent inject group and 22 patients (34 eyes) in the KDB group (Table 1). The mean follow-up period was 18 months for both groups. The mean age of the patients in both groups was similar (*p* = 0.873), and so was gender distribution (*p* = 0.174). In both groups, POAG was the most common type of glaucoma, accounting for 64/142 (45.1%) and 20/34 (58.8%) cases in the iStent inject and KDB groups, respectively. The other types of glaucoma were less common, but nonetheless mostly similarly distributed in both groups. Regarding severity, the proportions of mild, moderate, and severe glaucoma cases were also comparable in both groups. It can be noted that there was a slightly greater proportion of severe cases in the KDB group (19.4% vs. 13% for iStent), which is on the borderline of statistical significance (*p* = 0.052).

Pre-operative lens status showed a statistically significant difference between the two groups, with 139/140 (99.3%) and 31/34 (91.2%) phakic eyes in the iStent inject and KDB groups, respectively (*p* = 0.03). The mean visual field score was also better in the iStent group (−4.57 ± 5.5) than in the KDB group (−7.47 ± 6.6) (*p* = 0.04). In terms of visual acuity and IOP, no statistically significant differences were observed between the two groups. However, the mean number of glaucoma medications was significantly lower in the iStent inject group (1.82 ± 1.2) compared to the KDB group (2.55 ± 0.9) at the pre-operative baseline (*p* < 0.001). These findings suggest that the higher number of glaucoma medications in the KDB group may be related to the presence of more severe cases in this group. These baseline demographics and clinical characteristics of the study participants are detailed in Table 1. In summary, despite slight differences, the two subgroups still appear to be well-matched for comparison of surgical outcomes.

### 3.2. Intraocular Pressure Outcomes

**Figure 1:**
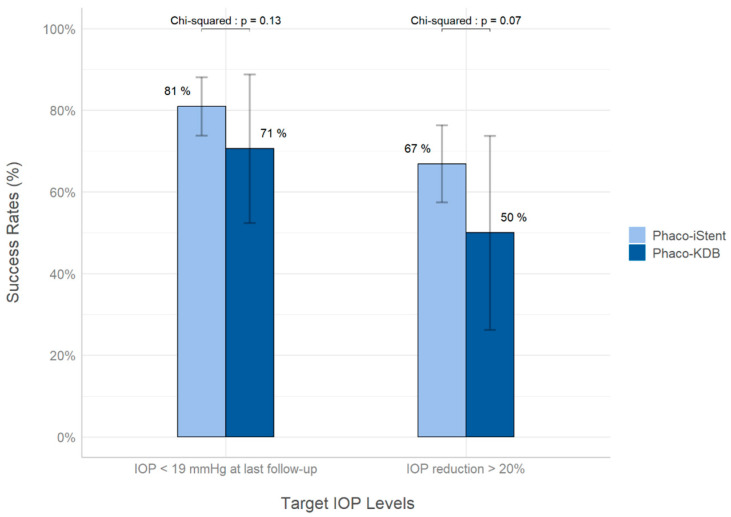
Success rates in achieving 20% IOP reduction and IOP ≤ 19 mmHg after MIGS combined with cataract extraction. The error bars represent 95% confidence intervals.

As shown in Figure 1, the iStent inject group had slightly better results in both primary outcomes although these differences were not statistically significant. In the iStent inject group, 67% of the patients achieved a 20% reduction in IOP compared to 50% in the KDB group (*p* = 0.07). Furthermore, 81% of the patients in the iStent inject group achieved an IOP under 19 mmHg at the last follow-up versus 71% in the KDB group (*p* = 0.13). The results suggest that KDB and iStent inject are both effective at reducing IOP and at achieving clinically significant results when combined with cataract extraction.

**Figure 2:**
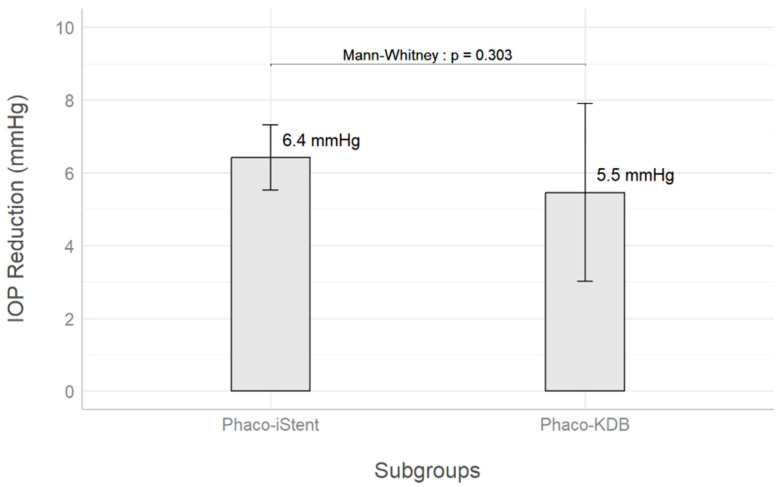
Average IOP reduction between KDB goniotomy and iStent inject groups. The error bars represent 95% confidence intervals.

In the KDB group, the mean IOP decreased from 22 mmHg to 16.5 mmHg at the last follow-up for a 5.5 mmHg average reduction (25% decrease). For the iStent inject group, mean IOP decreased from a baseline of 20.8 mmHg to 14.4 mmHg for a 6.4 mmHg reduction (31% decrease). These changes are illustrated in Figure 2. There was no statistically significant difference in mean IOP reduction between both groups (*p* = 0.30).

**Figure 3:**
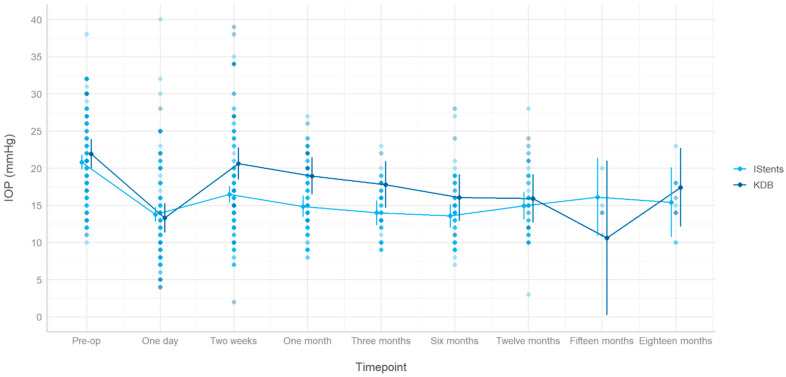
Average IOP at different follow-up time points for both groups. The follow-ups occurred at 1 day, 2 weeks, 1 month, 3 months, 6 months, 12 months, 15 months, and 18 months. The light blue circles represent IOP values of iStent patients, while the dark blue circles represent KDB patients. The error bars represent 95% confidence intervals.

For both subgroups, the patients’ post-operative IOP experiences an immediate drop at day 1. The IOP then peaks slightly at the 2-week mark (more in the KDB group than in the iStent group), before gradually decreasing in the following months and stabilizing. Although the iStent group appears to have lower IOP measurements at all time points, except at 15 months, the difference between the two groups was not statistically significant at any instance. Figure 3 demonstrates the evolution of the mean IOP in both groups throughout 18 months of follow-ups. The larger confidence intervals at the 15- and 18-month mark can be explained by the smaller sample sizes at those time points. As specified by our protocol, our inclusion criteria only required patients to have a minimum of 12 months of follow-ups. As such, many patients did not receive systematic follow-up appointments after 1 year, which significantly reduced the number of data points at 15 and 18 months.

Subgroup analysis was conducted for different types of glaucoma, including POAG, PXG, and PDG, as well as different levels of glaucoma severity (i.e., mild, moderate, and severe). In terms of IOP outcomes, our study results indicated that both KDB and iStent inject were equally effective in reducing IOP in all subgroups. When separated by glaucoma severity, patients’ IOP evolution patterns remained similar across all subgroups over 18 months (Figure S1). Patients in the iStent group often had lower mean IOP than their counterparts in the KDB group, but these differences are non-significant at the last follow-up in all severity subgroups. The IOP trends were also similar when patients were separated by glaucoma subtype (Figure S2). Apart from the exact IOP values that differed, all graphs displayed an initial drop at day 1, a rebound at 2 weeks, and a progressive decrease until the last follow-up.

### 3.3. Secondary Outcomes

**Figure 4:**
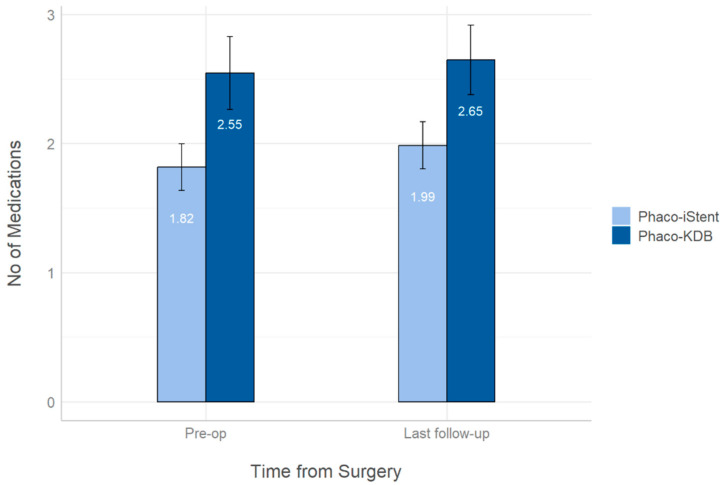
Number of IOP-lowering medications in both groups pre-operatively and at last follow-up. The error bars represent 95% confidence intervals.

Figure 4 shows the number of medications required by patients in the KDB and iStent inject groups pre-op and at the last follow-up. The number of medications does not decrease in either group from pre-op to the last follow-up.

As for visual outcomes, the mean BCVA in the iStent group improved from 0.25 ± 0.20 logMAR pre-operatively to 0.13 ± 0.17 at the last follow-up (*p* < 0.001). In that same time period, mean BCVA in the KDB group improved from 0.25 ± 0.24 logMAR to 0.16 ± 0.20 (*p* = 0.11). This amelioration of patients’ VA occurs in both groups, but only the changes in the iStent group are statistically significant. Additionally, there is no statistically significant difference in endpoint BCVA between the two groups (*p* = 0.38).

### 3.4. Adverse Events and Failure Rate

Both procedures were generally well tolerated, and adverse events, as displayed in Table 2, were mostly mild to moderate in intensity. The KDB group experienced a higher rate of adverse events (47.1%) than the iStent group (12.0%). The most common complications in the iStent group were rebound uveitis (6.3%) and ocular hypertension (OHT) (3.5%), while in the KDB group microhyphema/hyphema (29.4%), rebound uveitis (5.9%), and cystoid macular edema (CME) (5.9%) were the most frequent ones. CME and epiretinal membranes (ERMs) only occurred in the KDB group. We do not report the occurrence of any other complications during this study’s follow-up duration.

The failure criterion for this study was the need for subsequent filtration surgery during the follow-up period. The iStent inject group had 6 failures (4.2%), while its KDB counterpart had 10 failures (29.4%). The KDB group thus had a significantly higher failure rate than its counterpart (*p* < 0.001). These observations were similar among all types of glaucoma and all types of severity when multivariable analyses were conducted.

## 4. Discussion

### 4.1. Outcome Analysis

When compared to previous studies conducted by Arnljots and Economou in 2021 and by Barkander et al. in 2023, our study also found that both KDB and iStent inject produced a clinically significant reduction in IOP [33,35]. To our knowledge, those are the only previous investigations that evaluated the second-generation iStent. In terms of primary outcomes, Arnljots and Economou’s study found that KDB was more effective than the iStent in achieving a 20% IOP reduction (84% vs. 59%) [33]. Barkander et al. also suggest that KDB could be a slightly more effective procedure, with KDB successfully reducing IOP by 20% in 51% of patients compared to 46% for iStent injects [35]. However, in our study, it is the iStent that produced better results (67% vs. 50%). It is worth noting that these differences were not statistically significant in either study. It is relevant to consider that the difference in results between these studies can be attributed, at least in part, to differences in surgeon experience and preferences. In both previous studies, the KDB procedure was performed more frequently than the iStent inject procedure, while in our study, the iStent inject was performed more frequently than the KDB procedure. Surgeons who are more experienced with a particular technique may have a better understanding of its nuances, operate with higher degree of precision, and be more skilled at managing any potential complications. Therefore, it is important for ophthalmologists to select the surgery that they are most comfortable with and have the most experience in performing.

For combined KDB–phaco surgeries, the current literature reports IOP reductions ranging from 14% to 33% [13,33,35,36,37]. For combined iStent procedures, past investigations have demonstrated IOP reductions in the range of 14% to 35% [33,35,36,37,38]. For both techniques, the IOP reductions we report (25% for KDB and 31% for iStent) fall within these ranges and are hence consistent with previous findings. As for IOP measurements at various time points, the spike seen post-operatively at 2 weeks may be due to an overlapping steroid response that resolved on its own in the subsequent months with steroid cessation.

Both previous studies also reported a significant reduction in the number of glaucoma medications in both groups after surgery, and Barkander et al. found that reduction to be greater in their KDB group than in their iStent group [33,35]. In contrast, our study found no significant difference in the mean number of IOP-lowering medications from pre-operative to the last follow-up. In our cohort, patients in the KDB group also had on average more medications than those in the iStent group. This difference may be attributed to the KDB group having a greater proportion of severe glaucoma compared to the iStent group because the severity of the disease can affect the number of drugs required. Therefore, it cannot be concluded that KDB is less effective than iStent inject based solely on the number of medications used.

In terms of visual acuity, patients in both groups experienced BCVA improvements after surgery, though only the improvement in the iStent subgroup was statistically significant. Even though phacoemulsification was mainly performed to lower IOP, that procedure could have contributed to the BCVA changes as a confounding factor, especially in those who had clinically significant cataract. Arnljots and Economou also found BCVAs that showed a tendency towards improvement post-operatively. However, their changes were not statistically significant; this was potentially caused by their small number of participants [33]. In meta-analysis by Guedes et al. (2025) comparing phaco–KDB with phaco–iStent, the findings showed significantly higher surgical success rates in the KDB group than the iStent group over 12 months of follow-ups, which differ from our results [39]. The authors also found KDB to be more effective in lowering IOP than the first generation iStent. However, when compared with the second generation iStent, both MIGS displayed similar IOP reductions [39]. There were also no differences between both procedures in terms of mean BCVA changes [39].

### 4.2. Safety

In our study, a significantly greater rate of adverse events occurred in the KDB group (47.1%) than the iStent group (12.0%). Our findings concur with results from Lee and colleagues’ report in which complications, although uncommon, happened more frequently after KDB–phaco (27.3%) than iStent–phaco (3.4%) [29]. Lee et al. also described considerably higher rates of IOP increases in their KDB cohort (18.2% vs. 1.7%) [29]. In contrast, both groups in our investigation experienced statistically similar rates of OHT. As for other complications Barkander et al. reported a higher rate of hyphema in the KDB group with it occurring in 62% of eyes in that group compared to 14% in the iStent group [35]. Even though our rates of hyphemas are lower, our study still found hyphemas to be more frequent after KDB procedures. To assess whether this difference could be due to our KDB group having more severe glaucoma cases, a multivariable analysis comprising age at intervention, gender, lens status, type of glaucoma, and number of pre-operative glaucoma medications in both subgroups was conducted. Even in the subgroup analysis of only mild cases, we still observe a higher complication rate as well as a higher failure rate in the KDB group. Therefore, this observation cannot be simply justified by the fact that KDB patients had more severe glaucoma. As for iStent surgeries, one more aspect to consider is its permanent implantation of a metal device into the eye. As with other foreign objects, migration and obstruction may occur over time, even though none of those complications happened in our population during our study’s follow-up period [40,41]. Overall, our investigation suggests that KDB and iStent inject are safe, but KDB procedures seem to have a higher complication and failure rate. In Guedes et al.’s review, however, both surgeries had comparable rates of adverse events [39].

### 4.3. Strength and Limitations

One of the major strengths of our study is that it is, to our knowledge, the first in North America to directly compare the efficacy and safety of two different MIGS procedures: the KDB goniotomy and the second-generation trabecular micro-bypass stent. Prior studies frequently evaluated each of these procedures separately or examined the first-generation iStent. Thus, there have been few direct head-to-head comparisons until now. Our study attempts to enhance current insight on these surgeries by having a large sample size, which allows for more robust analyses. Furthermore, our study had a standardized surgical protocol with all surgeries performed by a single surgeon, which reduces the potential for variability in outcomes due to differences in surgical technique. Another strength of this study is that it includes patients with a broad range of glaucoma severity, including severe cases. This is in contrast to a previous report that only included patients with mild to moderate glaucoma [33]. Therefore, our study provides more comprehensive data on the effectiveness of both procedures in treating a wider range of glaucoma patients and increases its generalizability. However, it is important to note that the results of our study may not necessarily apply to more severe cases of glaucoma since the number of severe cases in our study was still relatively small.

Our study also has certain limitations that should be considered. Firstly, the retrospective design of our study increases the risk of selection bias and confounding factors due to its lack of randomization. While the pre-operative indications were similar between the two groups, allocating patients to either of the surgical techniques depended mainly on the surgeon’s preferences, a potential selection bias. Minor differences in baseline clinical characteristics are also present, with patients in the iStent group having a lower mean IOP and smaller numbers of medications despite these differences not being statistically significant. Secondly, our report has a relatively short follow-up period of 18 months, which limits its ability to evaluate the long-term efficacy and safety of these procedures. Additionally, our study was conducted at a single center, which may limit the generalizability of the findings to other populations and healthcare settings. Our study also did not evaluate patient-reported outcomes, such as quality of life, which could provide important insights into the overall impact of these procedures on patients. Furthermore, although our study included both patients with and without prior selective laser trabeculoplasty (SLT), we did not perform the corresponding subgroup analyses and are thus unable to comment on its impacts on IOP-lowering outcomes. In the literature, a recent retrospective cohort study by Mitchell and colleagues (2024) demonstrated that prior SLT status did not affect post-MIGS mean IOP after 36 months of follow-ups [42]. However, they noted that patients with prior SLT were more likely to undergo reoperation than those without prior SLT. The authors hypothesized that such patients had higher baseline IOP and more severe glaucoma, which could explain the higher risk of needing additional subsequent surgeries [42]. Lastly, our study lacked a control group that underwent cataract surgery alone, and it is important to acknowledge that cataract extraction alone is known to reduce IOP by 2–4 mmHg from baseline [43]. This nonetheless reflects routine clinical practice where iStent implantation and goniotomy with KDB are commonly performed together with cataract surgery. While the effects of the MIGS in our study cannot be isolated from that of cataract surgery, each group is equally affected, thus allowing meaningful comparisons between groups.

## 5. Conclusions

In summary, this study provides evidence that both KDB and iStent inject are effective in lowering IOP when combined with phacoemulsification. Both procedures achieved significant IOP reductions with no statistically significant difference between the two groups. Both techniques demonstrate a convincingly favorable safety profile without any sight-threatening complications although higher adverse events and failure rates were observed with the KDB procedure. Hence, the choice of procedure should be based on individual patient factors, including anatomy and disease severity, as well as surgeon preference and experience.

This study highlights the importance of ongoing research in MIGS for glaucoma, including larger studies with longer follow-up times to assess the safety, efficacy, and cost-effectiveness of different MIGS options. Larger randomized controlled trials are needed to confirm these findings and provide more robust evidence. Furthermore, additional studies may be necessary to investigate the long-term outcomes and cost-effectiveness of these procedures. Despite its limitations, this study contributes to the current knowledge base and may guide clinical decision-making for patients with coexisting cataract and glaucoma. Ultimately, the goal of glaucoma management is to preserve visual function and quality of life for patients, and MIGS represents an important and rapidly evolving field in achieving this goal.

## Figures and Tables

**Table 1:** Pre-operative baseline demographics and clinical characteristics.

Parameters		iStent	KDB	*p* Value
No. of eyes		142 (80.7%)	34 (19.3%)	
No. of patients		88 (80%)	22 (20%)	
Mean age (years)		75.5 ± 5.9	75.9 ± 6.1	0.57
Gender				
	Female	39 (44.3%)	12 (54.5%)	0.17
	Male	49 (55.7%)	10 (45.5%)	
Glaucoma diagnosis				
	POAG	64 (45.1%)	20 (58.8%)	0.79
	PACG	12 (8.5%)	3 (8.8%)	
	Mixed	11 (7.75%)	1 (2.9%)	
	PXG	2 (1.41%)	1 (2.9%)	
	PDG	2 (1.41%)	2 (5.9%)	
	NTG	21 (14.8%)	5 (14.7%)	
	OHT	30 (21.1%)	2 (5.9%)	
Severity				
	Mild	61 (66.3%)	13 (41.9%)	0.052
	Moderate	19 (20.7%)	12 (38.7%)	
	Severe	12 (13.0%)	6 (19.4%)	
Pre-operative lens status				
	Phakic	139 (99.3%)	31 (91.2%)	0.03
	Pseudophakic	1 (0.7%)	3 (8.82%)	
Mean BCVA (logMAR)		0.25 ± 0.20	0.25 ± 0.26	0.75
Mean visual field (MD)		−4.57 ± 5.5	−7.47 ± 6.6	0.04
Mean IOP (mmHg)		20.8 ± 6.0	22 ± 7.3	0.35
Mean number of glaucoma medications		1.82 ± 1.2	2.55 ± 0.9	<0.001

**Table 2:** Comparison of type and rate of post-operative adverse events between iStent and KDB groups.

Post-Op Adverse Events	iStent Inject	KDB	*p* Value
Rebound uveitis	9 (6.3%)	2 (5.9%)	1.00
Microhyphema/Hyphema	3 (2.1%)	10 (29.4%)	<0.01
OHT	5 (3.5%)	1 (2.9%)	1.00
CME	0 (0%)	2 (5.9%)	0.04
ERM	0 (0%)	1 (2.9%)	0.19
Total	17 (12.0%)	16 (47.1%)	<0.001

## Data Availability

Data is contained within the article.

## Supplementary Materials

The following supporting information can be downloaded at: https://www.mdpi.com/article/10.3390/biomimetics10100691/s1, Figure S1: Average IOP at different follow-up time points for both groups separated by glaucoma severity. The error bars represent 95% confidence intervals. Figure S2: Average IOP at different follow-up time points for both groups separated by glaucoma subtype. The error bars represent 95% confidence intervals.

## Abbreviations

The following abbreviations are used in this manuscript:

BCVA	Best-corrected visual acuity
CME	Cystoid macular edema
ERM	Epiretinal membrane
IOP	Intraocular pressure
KDB	Kahook Dual Blade
LogMAR	Logarithm of the minimal angle of resolution
MIGS	Microinvasive glaucoma surgery
NTG	Normal tension glaucoma
OHT	Ocular hypertension
PACG	Primary angle-closure glaucoma
PDG	Pigment dispersion glaucoma
Phaco	Phacoemulsification
POAG	Primary open-angle glaucoma
PXG	Pseudoexfoliative glaucoma
SLT	Selective laser trabeculoplasty
TM	Trabecular meshwork
VA	Visual acuity
