# Supplementary material for: Efficacy and Safety of Kahook Dual Blade Goniotomy and Trabecular Micro-Bypass Stent in Combination with Cataract Extraction
Source: Biomimetics (Basel). 2025 Oct 14;10(10):691. doi: 10.3390/biomimetics10100691 (PMC12564784; doi:10.3390/biomimetics10100691)
Supplement: Supplementary file 1 [file biomimetics-10-00691-s001.zip › biomimetics-3856385-supplementary.pdf]

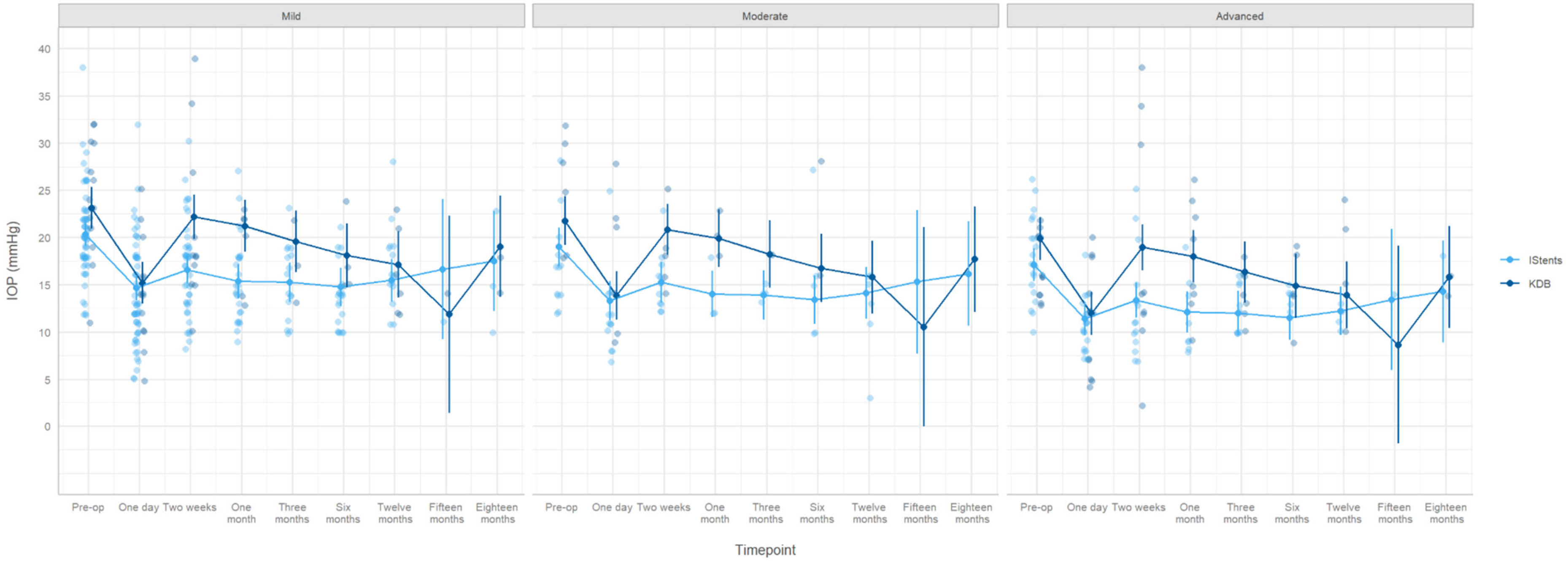

Figure S1. Average IOP at different follow-up times points for both groups separated by glaucoma severity. The error bars represent 95% confidence intervals.

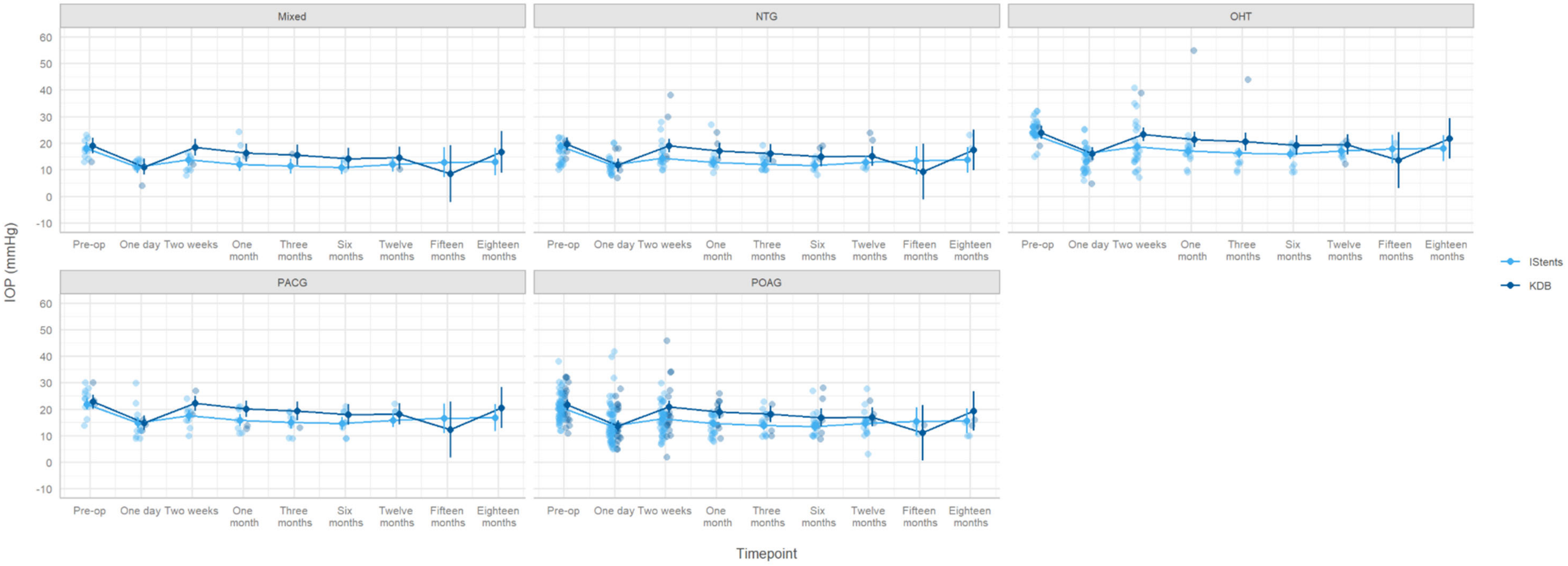

Figure S2. Average IOP at different follow-up times points for both groups separated by glaucoma subtype. The error bars represent 95% confidence intervals.
